# A Comparative Presentation of Mouse Models That Recapitulate Most Features of Alport Syndrome

**DOI:** 10.3390/genes13101893

**Published:** 2022-10-18

**Authors:** Stavros Nikolaou, Constantinos Deltas

**Affiliations:** 1 Biobank.cy Center of Excellence in Biobanking and Biomedical Research, University of Cyprus, Nicosia 2109, Cyprus; 2 School of Medicine, University of Cyprus, Nicosia 2109, Cyprus

**Keywords:** Alport syndrome, collagen IV, mouse models, kidneys, glomerular basement membrane, podocytes

## Abstract

Alport syndrome is a hereditary kidney disease caused by mutations in the three genes encoding for collagen IV: *COL4A3*, *COL4A4,* and *COL4A5*. Several mouse models have been created for the study of this disease with variable phenotypic outcomes. This review is an up-to-date presentation of the current mouse models existing in the literature with a detailed comparison of the phenotypic features characterizing each model. Although in humans it is primarily a glomerulopathy, data suggest that in some mouse models, the initial symptoms appear in the tubule-interstitial region rather than the glomerulus. Additionally, in some other models, the severity of disease in the tubule-interstitial region is affected by the genetic background. In conclusion, the phenotypic spectrum of each model appears to be affected by the model’s genetic background, the position of the genetic alteration within the gene, and the type of the genetic alteration. Despite these disparities, mouse models recapitulate with relatively high fidelity several features of the human disease, which makes them useful for studies aimed at better understanding cellular pathomechanisms and for finding new treatments.

## 1. Alport Syndrome

Alport syndrome (AS) was first described in a publication by Arthur C. Alport in 1927 while he was examining a British family. Members of this family suffered from familial congenital hemorrhagic nephritis [1]. One of the most prominent extrarenal symptoms described by Alport was nerve deafness of varying degrees. In addition to nerve deafness, blood and protein could be detected in their urine, at varying severity between family members. Affected members were also characterized by increased mortality due to end-stage renal disease (ESRD). Other extrarenal manifestations, which were later recognized by others in AS patients, were ocular abnormalities in the form of a dot and fleck retinopathy and anterior lenticonus [2,3,4]. Ultrastructurally, pathognomonic features of AS, seen on electron microscopy, are the thinning of the glomerular basement membrane (GBM) in early childhood, which later deteriorates to alternate thinning and thickening abnormalities as well as splitting and lamellation of the GBM, accompanied by podocyte foot process effacement. Depending on the type of mutation, and especially when there is the absence of collagen synthesis or secretion, it becomes obvious with negative immunofluorescent staining on light microscopy using specific anti-collagen IV alpha chain antibodies. Focal and segmental glomerulosclerosis may be another feature of light microscopy that complicates diagnosis.

AS presentation is clinically heterogeneous; in some cases, for various reasons including genotype–phenotype correlation, it appears early in life with patients progressing to ESRD before their 40s, while in other cases, it appears later in life (late-onset) with patients surviving with relatively normal or slightly impaired kidney function to old age [5,6]. The rarity of AS (prevalence estimated to be 1:5000 [7]) makes it difficult to drive conclusions about several epidemiological factors of AS, such as the age of onset.

AS is genetically heterogenous, caused by mutations in the genes encoding for trimeric collagen IV. Out of a total of six collagen IV alpha chains, α1-α6, only three configurations are biochemically feasible: α1α1α2, α3α4α5, α5α5α6. At maturity, the GBM contains the α3α4α5 trimer [8]. Collagen IV is the most abundant and critical component of the GBM, and mutations in any of the *COL4A3*, *COL4A4,* and *COL4A5* genes lead to AS pathology. According to older literature, most of the cases are caused by a mutation in the X-linked *COL4A5* gene (responsible for ~85% of the cases [7,9]). Mutations in the autosomal genes *COL4A3* and *COL4A4*, mapping head-to-head on chromosome 2q36.3, are responsible for the rest of the cases with autosomal recessive inheritance [7]. The X-linked type of AS is more frequent in women but more severe in men, while the autosomal form of AS occurs with equal frequency in men and women [10]. More recent data refer to later age of onset Alport-like or Alport spectrum phenotypes due to heterozygous mutations in the *COL4A3* or *COL4A4* gene, typically causing thin basement membrane nephropathy (TBMN) and presenting with microscopic hematuria since childhood. These patients have a variable risk of developing focal segmental glomerulosclerosis and progressing to chronic kidney disease, even ESRD, at ages ranging from 40 to 70 years. This phenotype is much more frequent but with incomplete penetrance, as about 1% of the population carries heterozygous *COL4A3/A4* mutations [5,11].

One of the hypotheses employed to explain the great variance in AS manifestation is the allelic heterogeneity between patients, as non-missense mutations (i.e., nonsense, indels, splicing defects) are accompanied by a greater risk of early-onset ESRD [6]. Similarly, missense mutations causing the substitution of glycine residues in the collagenous domain of the collagen alpha chains with amino acids having bulkier side chains also result in the earlier age of onset of ESRD [12]. Notwithstanding the role of allelic heterogeneity in interpreting the clinical phenotype, the likely role of genetic modifiers has also been implicated [13,14]. AS mouse models are a useful tool for eliminating this genetic heterogeneity bias, helping in the study of the syndrome in a more restricted and relatively more homogeneous genomic background while, at the same time, allowing us to design experiments involving higher and controlled genetic heterogeneity [15]. It does not escape our attention that other parameters, including environmental factors (i.e., temperature, light-dark cycle, food, water, and other mouse room conditions), might contribute to the clinical heterogeneity observed between the mice. We could not account for such factors in this review. Importantly, the use of AS mouse model enabled treatment experimentation that led to the repurposed use of ACE inhibitors, as modulators of the renin angiotensin aldosterone system (RAAS), with very positive results [16,17]. Because the genetics of mouse models differ from humans, the data derived from mice should be carefully interpreted. In this review, we focus on the mouse models created and studied for the understanding of the molecular basis of AS.

## 2. Mouse Models for Alport Syndrome

Several mouse models with mutations in *Col4a3*, *Col4a4,* and *Col4a5* are recorded in the Mouse Genome Informatics (MGI—http://www.informatics.jax.org (accessed on 25 September 2022)). Among these are models published individually as well as models created either by a private company (GemPharmatech Co., Ltd., Nanjing, China), the International Mouse Knockout Consortium (systematically phenotyping by the International Mouse Phenotyping Consortium—IMPC), and the Shangai Model Organisms Center—SMOC. In this review, we focus on the models published individually (including two models published but yet not recorded in the MGI). The reader can find relevant information about the phenotypes of the models not individually published from the corresponding websites (https://www.mousephenotype.org (accessed on 25 September 2022); https://www.modelorg.com/en/ (accessed on 25 September 2022) and https://www.gempharmatech.us/en/ (accessed on 25 September 2022)).

To date, 15 mouse models with genetic alterations at any one of the three collagen chains have been generated into different mouse strains (genetic backgrounds—see Table 1). The genetic alterations fall into six categories: gene knockouts, domain substitutions, nonsense mutations, mutations involving a splice donor site, missense mutations, and alterations substituting an amino acid with a small peptide. As a model, we consider a mouse carrying a specific genetic alteration in homozygosity or heterozygosity. Nine models carry alterations in *Col4a3*, three models carry alterations in *Col4a4,* and two in *Col4a5*. There is also one model with simultaneous alterations in both *Col4a3* and *Col4a4.* Among these, the most widely used are the *Col4a3* knockout models raising the possibility of experimental bias in the collected data. However, because each model has some unique characteristics, it is expected that other models will be used in research, depending on the hypotheses developed.

**Knockout**: Two knockout models have been created for the *Col4a3*. In both models, a *Neo* cassette has replaced a part of the NC1 domain of *Col4a3*. In the first model, the deletion involves the first three exons of NC1 (*Col4a3*^tm1Jhm^) [18], while in the second model, the deletion involves the exon 5 of NC1 domain (exons numbered from the carboxyl terminus—*Col4a3*^tm1Dec/J^) [19]. In addition to these models, a double knockout for both *Col4a3* and *Col4a4* has been created by insertion of the 4.1 kb tyrosinase minigene TyBS in between the two genes, deleting exons 1 through 12 of *Col4a4*, exons 1 and 2 of *Col4a3,* and the intergenic region comprising the promoter of the two genes (since currently there is not an official name of this model in MGD, we refer to this model as *Col4Δ3-4*) [28].

**Domain substitution**: One model has been created by a targeted knockin approach that has substituted the mouse Col4a3 NC1 domain by the human Col4α5 NC1, thus preventing the in vivo formation of the α3α4α5 collagen IV (*Col4a3*^tm1.1Rk1^) [21].

**Nonsense mutations**: Three models (one for *Col4a4* and two for *Col4a5* genes) were created by the introduction of nonsense mutations. In *Col4a4*, the mutation was introduced by ENU-mediated mutagenesis, and it involves glycine at position 400 (p.G400X; *Col4a4*^m1H^) [27]. In *Col4a5*, both alterations are targeted mutations, and they correspond to human-derived mutations. The older model was created by single nucleotide mutagenesis, mutating glycine at position 5 of exon 1 of the 7S domain (p.G5X; *Col4a5*^tm1Yseg^) [29]. The most recent model was created by a CRISPR/cas approach introducing a nonsense mutation at arginine 471 (p.R471X; *Col4a5*^em1Keha^) [30].

**Mutations causing splicing alterations**: Two models are created by mutations involving a splice donor site of the *Col4a4*. The first model was created by ENU-mediated mutagenesis of the +1 guanine to adenine, destroying the splice donor site of intron 8 (*Col4a4*^m1Btlr^). The mutation resulted in a frameshift error and a premature stop codon located in the collagenous domain of *Col4a4* [25]. The second model emerged as a spontaneous mutation of the +1 guanine to adenine in the splice donor site of exon 30 (*Col4a4*^bwk^). The mutation resulted in exon skipping but maintained the open reading frame. It does not lead to 100% alternative splicing as a large subset of mutant transcripts retain intron 30 [26].

**Missense mutations**: Three models carry the same missense mutation at *Col4a3* in a homozygous, heterozygous, and compound heterozygous condition. These models were created by site-directed mutagenesis aimed at the substitution of glycine at position 1332 into glutamate, thus recapitulating the corresponding human founder mutation COL4A3-p.Gly1334Glu. Since currently there is no official name for this model in MGD, we refer to this model as *Col4a3*^p.G1332E^. The compound heterozygous mouse was generated by crossing the knockin mouse with the previously established knockout mouse, *Col4a3*^tm1Dec/J^. Here we present in detail only the phenotype of the homozygous knockin (*Col4a3*^p.G1332E^) [22,23]. The compound heterozygous mouse has a similar phenotype, while the heterozygous presents TBMN with a reduced life span [22].

**Alterations substituting an amino acid with an octa-peptide**: Two models (a homozygous and a heterozygous one; *Col4a3*^em1Bghn^) have been created by a CRISPR/cas-mediated substitution of His1669 of exon 52 of *Col4a3* with a human-derived peptide of eight amino acids (QQNCYFSS; named as Zurich appendage) [24].

## 3. Comparison of Lifespan

All models have increased mortality compared to the wild-type controls of matching genetic backgrounds. Increased mortality is dependent on the type of mutation and the location within the corresponding gene. It is also affected by the gene dosage as haploinsufficiency affects lifespan less drastically compared to homozygosity. In addition, the model’s genetic background has been shown to be an essential determinant of the lifespan [31].

Both *Col4a3* knockouts live for approximately 2.5–3 months (50% survival of *Col4a3*^tm1Dec/J^ in a 129X1/SvJ background is 70.9 +/− 6.0 days; n = 20) [18,19,20]. Heterozygosity of *Col4a3*^tm1Dec/J^ extends lifespan almost 10-fold (50% survival 21.7 +/− 2.5 months; n = 10 as compared to 30.3 +/− 2.4 months in the wild type) [20]. Substitution of the mouse Col4a3 NC1 domain with the human Col4a5 NC1 domain also results in increased mortality as *Col4a3*^tm1.Rk1^ mice survive for 23–30 weeks in the C57BL/6J background (~6–7 months; n = 11) [21]. Interestingly, these authors have used the *Col4a3*^tm1Dec/J^ mice as positive controls after transferring in the C57BL/6J background (as opposed to the 129X1/SvJ used in the original study). They report that *Col4a3*^tm1Dec/J^ lifespan in the C57BL/6J background is increased to 6–7 months instead of 2-3 in the 129X1/SvJ.

All three missense models (*Col4a3*^p.G1332E^) are also characterized by an extended lifespan with mean survival time in homozygosity of 15.1 months (n = 31), in heterozygosity of 17.9 months and in compound heterozygosity of 16.07 months while wild-type mice in this study lived on average for 22.7 months (n = 23) in a mixed 129X1/SvJ—C57BL/6J background (initially in C57BL/6J, then backcrossed in 129X1/SvJ for five generations) [22]. The same mutation on the C57BL/6J background has not been studied in detail, but apparently, it does not significantly affect the mouse lifespan (Deltas C et al., unpublished results). It is known that the C57BL/6J background is protective of Alport-caused kidney dysfunctionality [27,32].

The lifespan of *Col4a3*^em1Bghn^ is not reported, although the heterozygous model (*Col4a3*^em1Bghn^/+) survives for at least one year [24].

Models carrying alterations in the *Col4a4* gene are also characterized by increased mortality: *Col4a4*^bwk^ has a mean age at death of approximately 3 months (84 days) [26] while *Col4a4*^m1Btlr^ lives for 6-7 months (approximately a two-fold increased survival) [25]. While this difference might be due to the different position of the mutation in the gene [33], it is also possible that prolonged lifespan in the *Col4a4*^m1Btlr^ is caused by the different background between the two models (DBA/2J vs. C57BL/6J). This can be clearly seen in the third model, *Col4a4*^m1H^, carrying a *Col4a4* truncating mutation, p.G400X. This mutation mimics a knockout model, as verified by the absence of the Col4a4 protein. When p.G400X is introduced in a C57BL/6J-enriched background, the mean survival is approximately 3 months (97 days), while when the same mutation is introduced in a C3H.Pde6b-enriched background, the mean survival is reduced to half (47 days) [27].

*Col4Δ3-4* (lacking the expression of both *Col4a3* and *Col4a4*) is also characterized by a similar lifespan to the previously described models as it survives for approximately 3 months (10–14 weeks—in the FVB/N background) [28].

Models carrying *Col4a5* alterations have a slightly increased survival compared to the rest. For example, male *Col4a5*^tm1Yseg^ mice have a median survival of approximately 5–6 months (23 weeks—C57BL/6J background). Female carriers of the same mutation have longer median survival (39 weeks) due to random X-inactivation [29]. Similarly, hemizygous male *Col4a5*^em1Keha^ mice start dying at 6.5 months (26 weeks; 72.2% died by 30 weeks—C57BL/6J background) [30]. It is unclear yet whether this is due to the genetic background or due to the affected gene, but the prolonged survival of the *Col4a4*^m1Btlr^, which is also into a C57BL/6J background, makes it possible that the genetic background is the factor affecting the prolonged survival of *Col4a5*-based models [25].

## 4. Comparison of Sex, Morphological, and Physiological Characteristics

There is a sparsity of data regarding epidemiological variables such as sex, mouse weight, kidney appearance, volume of drunk water, and volume of urine secreted. These are important variables as they can provide a sense of the initiation and progression of kidney dysfunction.

Although sex bias in the severity and progression of the disease is expected for the two *Col4a5* models due to their X-linked nature, such bias is not expected for the autosomal *Co4a3-* and *Co4a4*-based models. However, the phenotype of another autosomal kidney disease, polycystic kidney disease, is presented in a sex-dependent manner, despite its autosomal nature [34]. In addition, sex and background-dependent differences in the composition of GBM, associated with albuminuria, are reported in healthy mice [31]. The possible sex-dependent bias in disease manifestation has been examined using the *Col4a3*^tm1Dec/J^, and no difference was observed in disease progression [35]. In contrast, data derived from the *Col4a4*^bwk^ suggest a sex-dependent difference in the albumin-to-creatinine ratio [26]. This difference could be due to the different genetic backgrounds in the two studies [33], or it might be the result of the early mortality of these models masking a possible late-onset sex bias in disease manifestation. Since the early signs of disease in *Col4a3*^tm1Dec/J^ are already visible by the time these mice enter puberty (~1 month of age, see Table 2) [36], a sex bias cannot be excluded. Importantly, recent findings suggest a sex-biased difference in gene expression of more than 700 genes in the proximal tubules [37]. Thus, a model with longer survival, such as *Col4a3*^p.G1332E^, might be necessary to test this hypothesis. Thus far, no sex difference in lifespan has been reported for this knockin model.

Kidney coloration becomes pale in all models. *Col4a3*^tm1Jhm^ kidneys have a shrunken and wrinkled surface, while *Col4a5*^tm1Yseg^ kidneys have surface pallor, dullness, and pockmarking [18,29]. A smaller kidney size is reported for *Col4a3*^tm1Jhm^, *Col4a3*^tm1Dec/J^, *Col4a4*^m1H^, *Col4a5*^em1Keha^ and in *Col4a4*^m1Btlr^ [18,19,25,27,30]. Similar observations are also made by Odiatis and Deltas for the *Col4a3*^p.G1332E^ (unpublished results). In contrast, *Col4Δ3-4* kidneys are approximately 15% larger in size than their control littermates [28]. The reason for this difference is still unknown, and since then, no other model has been described with kidney size larger than controls. At the ESRD, there are reports of a rough granular appearance [19]. Cystic appearance, which reminds of polycystic kidney disease, was reported in patients heterozygous for *COL4A3/4* mutations [38,39].

Uremic cachexia is prevalent in chronic kidney disease and is associated with increased mortality [40,41]. Six models are reported to be cachectic. For example, *Col4a3*^tm1Jhm^ is characterized by body weight reduction starting after 2 months [18]. Similar findings are reported for the *Col4a3*^tm1Dec/J^ and the *Col4a4*^bwk^ [26,35]. *Col4a4*^m1H^ is characterized by a reduced weight at approximately 1.5 months (5 weeks). Body weight of hemizygous *Col4a5*^em1Keha^ males appears normal until 14 weeks of age with a slower gain as compared to wild-type mice after 16 weeks [30], but when examined at 24 weeks, the body weight of these mice is reduced compared to the wild type [42]. In the *Col4a3*^p.G1332E^, body weight is gradually decreased from 6 months onward (Odiatis and Deltas—unpublished results). In contrast to these observations, the body weight of *Col4a3*^tm1.1Rk1^ mice is normal. Although the authors do not define this, this unexpected finding might be due to an early age of examination [21].

Data derived from only two models (*Col4a3*^tm1Jhm^ and *Col4a4*^m1H^) report an increased urine volume (increased after 2 months in the *Col4a3*^tm1Jhm^ and after 1 month in the *Col4a4*^m1H^) [18,27]. No other data exist regarding the urine volume excreted by Alport mice.

The effect of Alport on the volume of consumed water was examined only in the *Col4a4*^m1H^. The researchers report an increase in consumed water volume at one month (4 weeks), which agrees with the observation that urine volume in this model is also increased at this time [27].

## 5. Comparison of Homeostasis and Metabolism

Proteinuria is a common symptom of all Alport mouse models. In the *Col4a3*^tm1Jhm^, protein/creatinine ratio increases from 2- to >20-fold after two months [18]. Similar findings apply to the *Col4a3*^tm1Dec/J,^ which develops proteinuria (10–15 mg/mL) by the age of 1.5–2 months in the 129X1/SvJ background [19]. The half dosage of *Col4a3* in the heterozygous *Col4a3*^tm1Dec/J^ develops mild proteinuria (>0.1 g/L) at 3 months, which is increased to >3 g/L before death [20]. *Col4a3*^tm1.1Rk1^ is presented with an elevated albumin-to-creatinine ratio by the age of 12 weeks (17.37 ± 0.83). Importantly, these researchers have used the *Col4a3*^tm1Dec/J^ as a positive control, albeit in a C57BL/6J background. They report an elevated albumin-to-creatinine ratio in their positive control by the age of 12 weeks (10.36 ± 0.99), emphasizing the significance of the background on proteinuria initiation [21]. Mild proteinuria also appears in the knockin *Col4a3*^p.G1332E^ at 3 months (>0.1 g/L) which is increased more than 10-fold by the age of 12 months (>1 g/L) as well as in the *Col4a3*^em1Bghn^ (between 9 and 23 weeks of age) and the heterozygous *Col4a3*^em1Bghn^/+ (at one year of age) [22,24]. The *Col4a4*^m1Btlr^ is also characterized by proteinuria (measured by Chemstrip analysis) that initiates at 3 months (3/23 mice > 30 mg/dl) and is established by the age of 4 months (7/7 mice > > 30 mg/dL) [25]. The *Col4a4*^bwk^ is characterized by an elevated albumin-to-creatinine ratio by 4 weeks (reaching 3400 mg/g at 6 weeks). Proteinuria in the *Col4a4*^m1H^ appears at 4 weeks when in a C3H.Pde6b-enriched genetic background [27]. In the *Col4Δ3-4*, proteinuria is detectable by the age of 2 weeks (elevated 10-fold at the age of 1 month) [28]. Proteinuria is also detected in both *Col4a5*-based models: in the *Col4a5*^tm1Yseg^ model is detected in hemizygous males older than 7 weeks and in female carriers older than 9 weeks [29], while in the *Col4a5*^em1Keha^ urinary albumin in males increases after 6 weeks and remains high after 22 weeks (mean: 2,108 ± 608 μg/16 h—only male mice were used in this study) [30].

In contrast to proteinuria, hematuria is not a consistent feature in Alport mice. While some models appear to be negative for hematuria, in others, it is present. A subset of those models where it is positive demonstrates intermittent presentation. For example, the *Col4a3*^tm1Jhm^ does not develop hematuria, while the *Col4a3*^tm1Dec/J^ develops microscopic hematuria at 2 weeks, although both are knockout models [18,19]. The heterozygous *Col4a3*^tm1Dec/J^ also presents hematuria early in life (after 2 months—>10 erythrocytes per field of view x400) [20]. The *Col4a3*^p.G1332E^ is the only model characterized by intermittent hematuria among the *Col4a3*-based models, which develops after 3 months [22]. Among the *Col4a4*-based models, only dipstick data exist regarding hematuria. Thus, in the *Col4a4*^bwk^, no hematuria is detected, while hematuria appears in the *Col4a4*^m1Btlr^ at the age of 3 months [25,26]. Hematuria also appears in the *Col4a4*^m1H^ at 4 weeks when in a C3H.Pde6b-enriched genetic background [27]. Data for the *Col4a5*-based models exist only for the *Col4a5*^em1Keha^, where hematuria was detected after 22 weeks of age) [30]. Hematuria was not measured in the *Col4a3*^tm1.1Rk1^ nor in the *Col4a3*^em1Bghn^, although Alport patients carrying the Zurich variant are characterized by hematuria [21,24].

A rise in the blood urea nitrogen levels (BUN) and serum creatinine can be seen in all models, sometimes shortly before their death. In the *Col4a3*^tm1Jhm^, BUN and serum creatinine are initially maintained at normal levels but are increased after two months of age [18]. Similar data are reported for the *Col4a3*^tm1Dec/J^ model, with BUN beginning to rise at 10 weeks and eventually reaching 10-fold the wild-type levels [19]. Serum creatinine in the *Col4a3*^tm1.1Rk1^ is increased 4-fold at 22 weeks, reaching 0.425 ± 0.0692 mg/dL [21]. In the heterozygous *Col4a3*^tm1Dec/J^, BUN begins rising much later, at 18 months, and eventually exceeding 125 mg/dL before death [20]. This finding agrees with data reported for the *Col4a3*^p.G1332E^, where both serum urea and serum creatinine were increased at 15–22 months in most animals [22]. In the *Col4a4*^m1Btlr^, BUN is only elevated at 5 months but not at 4 months, while in the *Col4a4*^m1H^, both BUN and creatinine are raised already by the first month [27]. In the *Col4Δ3-4*, BUN is reported to be normal at 6 weeks, but it was 10-fold elevated at 12 weeks [28]. Increased BUN and serum creatinine can also be detected in the *Col4a5*^tm1Yseg^ at 16-week-old hemizygous male mice [45].

## 6. Comparison of Glomerular Alterations

A hallmark of AS is the characteristic thinning and splitting structure of the GBM [46]. Therefore, all Alport mouse models should share similar glomerular lesions, and this seems to be true, albeit with a background-dependent difference in the timing and severity of these alterations [31].

The *Col4a3*^tm1Jhm^ develops a basket-weave-like GBM with altered composition (abnormal presence of perlecan, fibronectin, and collagen VI at 60 days). In addition, electron microscopy analysis shows that the GBM of this model is decellularized, rough, and blebbed at 12 weeks [47]. The glomerulus of this model has a thickened Bowman’s capsule as well as thickened and closed capillary loops filled with hyalin. Immunofluorescence analysis verified the absence of Col4a3, Col4a4, and Col4a5 and documented the expression of Col4a1 and Col4a2 proteins in the GBM [18]. A similar finding is reported for the *Col4a3*^tm1Dec/J^. This model has an altered appearance of the GBM with focal thinning and splitting, progressing rarefication, and multilamination from peripheral to internal capillaries. GBM’s composition is also altered with increasing concentration of laminin-1 and ectopic expression of fibronectin and HSPG from 4 to 14 weeks. Bowman’s capsule is thickened, and there is an expansion of the mesangial matrix. Podocytes have an altered morphology with the appearance of microvilli on their pedicles. Col4a3, Col4a4 and Col4a5 are absent but there is ectopic expression of Col4a1 and Col4a2 into the GBM [19]. In the heterozygous *Col4a3*^tm1Dec/J^, it takes much longer for the GBM to be altered, and the effect is milder: only a reduction in its thickness is observed at 12 months, presenting typical thin basement membrane nephropathy. There is an occasional thickening of the Bowman’s capsule, and the mesangial cell number is increased. At 30 weeks (~7 months old), there is an ectopic expression of laminin-1 and fibronectin in the intra- and peri-glomerular space. In addition, signs of glomerulosclerosis and inflammation are present (infiltration by interstitial myofibroblasts and macrophages) [20].

The GBM of the *Col4a3*^tm1.1Rk1^ is also characterized by focal thinning and thickening at 22 weeks, and additionally, the collagen network structure is altered, composed by hexamers of Col4a1 and Col4a2 assembled into the α2α1α1/α2α1α1 network. Col4a4 protein is not expressed, and Col4a5 and Col4a6 expression are expanded into the mesangial area. Although Col4a3 is expressed as a hybrid mouse/human protein, it does not contribute to network formation. At the age of 12 weeks, there are signs of glomerulosclerosis initiation, which becomes extensive by the age of 22 weeks. At the same age, podocytes are characterized by foot process effacement [21].

GBM thinning and splitting are also seen in the knockin *Col4a3*^p.G1332E^ at the age of 5 months. These mice demonstrate podocyte foot process effacement and segmental or global glomerulosclerosis when examined at 20 months. In this model, a cleaved C-terminal fragment of collagen IV (including the NC1 domain—35 kDa in size), instead of the intact collagen IV, can be detected in the GBM already by the age of 4 months [22]. The researchers showed Mmp9 activation in the mutant glomeruli at approximately the same age, but the exact mechanism regulating the formation of the fragment and its role in disease remains unknown.

Although Col4a3, Col4a4, and Col4a5 protein chains are expressed in the glomeruli of *Col4a3*^em1Bghn^ mice (both the homozygous and the heterozygous models), the presence of the Z-appendage results in the abnormal structure of GBM. This includes thinning and thickening, lamellation, and, occasionally, splitting, along with podocyte foot process effacement. In addition, varying degrees of glomerulosclerosis with rare crescents are observed [24].

A similar phenotype is observed among all *Col4a4*-based models. *Col4a4*^bwk^ is characterized by synechia, expansion of the mesangial matrix, and glomerular crescents at the age of 3 months. In addition, this group reports the presence of glomerulosclerosis. A low expression of Col4a3, Col4a4, and Col4a5 is observed, which are still capable of assembling into protomers. In addition, increased expression of Col4a2 in the GBM is observed at 5 and 10 weeks. This model is also characterized by splitting, thickening, and basket-weave-like morphology of GBM at 6 and 9 weeks [26]. *Col4a4*^m1Btlr^ shows only occasionally increased periodic acid-Schiff staining in glomeruli at 2 months, but by the age of 5 months, the glomeruli develop focal and segmental glomerulosclerosis [25]. In the *Col4a4*^m1H^ model, there is an enlargement of glomeruli as well as increased cellularity, examined at 53 days [27]. The absence of Col4a4 was verified by immunofluorescence staining.

Glomeruli of the *Col4Δ3-4* are characterized by hyperplasia of parietal epithelial cells (thickened Bowman’s capsule) by the age of 5 months, increased number of mesangial or endocapillary cells, and increased cell proliferation, as determined by BrdU positivity, already by 4 weeks. Based on the location of the BrdU signal, increased proliferation is not detected in the podocytes. Crescentic glomerulonephritis is also observed. GBM is thin and duplicated at 2 weeks, and later it becomes thicker with basket weaving of the lamina densa and lacks expression of Col4a3, Col4a4, and Col4a5. Abnormally strong ectopic expression of Col4a1 and Col4a2 chains are detected in the capillary loops of the GBM [28].

In hemizygous *Col4a5*^tm1Yseg^ male mice, glomeruli at 4 weeks present with subtle findings such as capillary wall thickening and mesangial hypercellularity only to worsen at 7 weeks with capillary loop dilation/simplification, capillary tuft collapse, and capsular adhesions. Focal glomerulosclerosis is detected at 17 weeks. In these mice, GBM is characterized by lamellation at 4 weeks, which progresses into splitting at 17 weeks. Podocyte morphology is also altered with foot process effacement and vesiculation. The expression of *Col4a5* and *Col4a3* is lost, but the expression of *Col4a1* and *Col4a2* is maintained. In addition, the expression of *Col4a6*, normally detected within Bowman’s capsule, is also lost [29].

Glomeruli of *Col4a5*^em1Keha^ male mice present with subtle findings at 4 weeks, only to worsen later, by 6 weeks, with parietal cell hyperplasia/collapsed glomerular tuft in a few glomeruli and occasional foot process effacement associated with abnormal GBM. At this age, GBM is characterized by focal irregularity/thickening that is accentuated and becomes widespread from 14 to 30 weeks. At the same period, increasing areas of cellular crescents with fibrinoid exudate appear. These crescents are derived from two different cell types in the Bowman’s capsule: migrating CD44+ fibroblasts and α-Sma+ parietal endothelial cells. When examined at 22 weeks with low vacuum scanning electron microscopy (LVSEM), the GBM surface appears ragged in the mutant mice instead of smooth in the wild type. At this advanced age, there is widespread glomerular tuft collapse with thickened Bowman’s capsule, parietal cell hyperplasia, increased mesangial matrices, and increasing glomerular enlargement in parallel with increasing glomerulosclerosis. Expression of *Col4a5* is not detected either in glomeruli or tubules [30,42].

## 7. Comparison of Tubular Alterations

Defects in tubules are common among Alport mice, and, in general, the different mouse models share similar tubular alterations. Col4a3/α4/a5 chains are expressed in the mouse proximal tubular basement membrane, assuming the collagen IV-α3α4α5 configuration, but not in humans [48,49,50].

The *Col4a3*^tm1Jhm^ is characterized by atrophied and dilated tubules that are filled with hyalin at 60 days) [18]. A later study of the same model by other investigators reports the upregulation of the kidney injury molecule-1 (KIM-1—marker of injured proximal tubules) in the proximal tubules as well as increased apoptosis (detected by TUNEL assay) [43]. Such morphological tubular alterations are not reported in the original publication of the *Col4a3*^tm1Dec/J^, but these authors report the expression of Col4a1 and Col4a2 on the TBM and the enhanced staining for fibronectin on the walls of the tubules [19]. A more recent study of *Col4a3*^tm1Dec/J^ reports KIM-1 upregulation, increased cellular proliferation (shown by EdU incorporation), and increased levels of free cholesterol in the tubular cells of these mice [44]. Moreover, the culture of isolated primary *Col4a3*^tm1Dec/J^ tubular cells has shown that these cells are metabolically defective, as is shown by the defective mitochondrial respiration [44]. Heterozygosity of *Col4a3*^tm1Dec/J^ also has a negative effect on tubular cell physiology at the age of 12 months, as the tubules are filled with protein casts, and tubular cells are increased in size with vast amounts of euchromatin, suggesting an altered transcriptional profile [20]. For the *Col4a3*^tm1.1Rk1^, the only tubular alteration that is reported is tubular atrophy [21]. In the homozygous knockin *Col4a3*^p.G1332E^ model, immunofluorescence-based analysis shows severely impaired secretion of Col4a3, Col4a4, and Col4a5 in the tubular basement membrane, while expression of the α1 and α2 chains was normal [22]. About one-third of the mice showed a 75% degree of tubular injury.

In the *Col4a4*^m1Btlr^, tubular defects initiate before the age of 2 months as there are some dilated tubules by this time that progress into a more severe phenotype with tubular atrophy and dilation by 5 months [25]. In the *Col4a4*^bwk^, the existence of tubular protein casts and tubular atrophy is reported at 3 months when the mutated *Col4a4* is in a NONcNZO4/Lt genetic background. The effect of this mutation on tubules is background-dependent because there is an earlier-onset of tubular phenotype when the mutation is in DBA/2J than when it is in 129S1/SvImJ (6 weeks instead of 9 weeks of age) [26]. Such difference in the onset of phenotype is not observed in glomeruli of the same mice suggesting a differential effect of the genetic background on glomeruli and tubules. Tubular dilation and hyaline casts are also observed in the *Col4a4*^m1H^ model [27]. However, this group has also shown that in their model, tubular defects precede glomerular defects when in C57BL/6J-enriched genetic background. For example, mild tubular defects can be seen in the C57BL/6J-enriched genetic background by the age of 7 weeks, while no glomerular defects can be seen at the same age in the same background, suggesting that the genetic background not only can determine the severity and progression of symptoms on the tubules, but it can also determine the tissue (glomerulus or tubules) where the onset of disease will be initiated.

In the *Col4Δ3-4* model, tubular cells become more proliferative at the age of 4 weeks, as is shown by BrdU positivity. There is also tubular injury that progresses to tubular atrophy by the age of 8 weeks [28]. The authors did not examine tubular cell alterations in the two *Col4a5*-based models [29,30].

## 8. Comparison of Fibrosis and Inflammation in the Interstitium

Fibrosis and inflammation are common aspects of all models developing late during disease progression. In the *Col4a3*^tm1Jhm^ mice, there is a deposition of extracellular matrix between tubules by the age of 60 days [18]. A similar finding is reported for the *Col4a3*^tm1Dec/J^ knockout model. In this case, the tubular walls show enhanced staining with fibronectin by 14 weeks, and additionally, there is extensive fibrotic cell proliferation, as shown by EdU incorporation, as well as lipid accumulation in the interstitial space. Increased expression of the macrophage marker CD68 suggests increased macrophage infiltration and inflammation [19,44]. Fibrosis is also detected in the heterozygous *Col4a3*^tm1Dec/J^ as well as the *Col4a3*^tm1.1Rk1^ models, albeit at an older age (upregulated expression of the profibrotic cytokines TGF-β1 and CTGF at 10 and 30 weeks in the heterozygous *Col4a3*^tm1Dec/J^ and positivity for periodic acid-Schiff and Masson Trichrome staining at the age of 22 weeks for *Col4a3*^tm1.1Rk1^ [20,21]). The *Col4a3*^p.G1332E^ model also develops moderate to severe periglomerular and interstitial fibrosis when examined at 20 months, shown by upregulation of TGF-β1 and Acta2 in glomerular and kidney lysates as well as by histochemical staining with periodic acid-Schiff staining, Sirius Red, and Masson’s Trichrome [22].

*Col4a4*^m1Btlr^ develops interstitial fibrosis and collagen positivity from the cortex into the medulla by the age of 5 months [25]. In the *Col4a4*^bwk^, there is tubulointerstitial nephritis and inflammatory cell infiltration (derived from the innate immune system) in the interstitium at the age of 3 months when the mutation is on the NONcNZO4/Lt background [26]. This group has shown that the initiation of nephritis and inflammation in the interstitium is also background-dependent because when the mutation resides on the DBA/2J background, inflammatory cell infiltration in the interstitium occurs as early as 6 weeks, while when the mutation resides on the 129S1/SvImJ background, inflammation is delayed (at 9 weeks of age) [26]. Similar findings are reported for the *Col4a4*^m1H^ model: fibrotic and inflammation markers are reduced at the age of 4 weeks in a B6-enriched model compared to a C3H-enriched one suggesting an effect of genetic background into the initiation of fibrosis and inflammation as is also the case for glomerular and tubular alterations [27].

In the *Col4Δ3-4*, interstitial fibrosis is apparent at 8 weeks. Fibrosis and inflammation are also detected in the *Col4a5*^tm1Yseg^ model. In addition, macrophage infiltration (as shown by F4/80 positivity) and expression of pro-inflammatory cytokines such as Il-6, KC/Il-8, Stat3, and Socs3, are detected in the kidneys of 16-week-old *Col4a5*^tm1Yseg^ mice (C57BL/6 background) [45]. Moreover, these mice are characterized by the expression of the pro-fibrotic genes of *a-SMA*, *Tgf-b,* and *Col1a1*, as well as *Mmp9* [45]. The *Col4a5*^em1Keha^ develops tubulointerstitial changes associated with glomerulosclerosis at 22 weeks [30].

## 9. Comparison of Ocular and Hearing Defects

Kidney defects are not the sole defect caused by AS. Hearing loss and ocular abnormalities are frequently observed in patients. Although in mouse models of AS, this field is poorly studied, the current data suggest that auditory and ocular defects appear in Alport mice, albeit not with 100% penetrance, and are model-dependent (see Table 3). In the *Col4a3*^tm1Jhm^, 3 out of 12 mice (at the age of ~3 months) had defective auditory sensitivity as determined by the auditory brainstem response (ABR) test [18]. In the *Col4a3*^tm1Dec/J^ model, only sporadic results are reported by the same test, but an irregular shape of the basement membrane encasing the anterior lens is also reported [19]. A more detailed study of these mice reports severe structural and biochemical alterations in the cochlear basement membranes, endothelial cell swelling, a decrease in internal capillary diameter, and an increase in ABR between 6 and 8 weeks [51]. The *Col4a3*^tm1Dec/J^ model was also studied by others, who also reported an elevated hearing threshold, as measured by ABR, and reduced anterior capsule apical angle (ACAA) in the eye. In addition, these researchers report the increased thickness of both the cochlea basement membrane and the basement membrane of the retinal capillaries [44]. AS patients carrying the Zurich variant are characterized by hearing loss, but whether the mouse models carrying Z-appendage are also characterized by hearing loss is still unknown [24].

In *Col4a4*^bwk^, no deviation in hearing is reported, nor alteration in eye histology [26]. Similar findings are reported for the *Col4a4*^m1H^ model (no difference in ABR between wild type and mutants at the age of 6–7 weeks; no alterations in optokinetic drum scores, slit lamp, and ophthalmoscopic observations at the age of 6–7 weeks [27]). In contrast, 2 out of 6 *Col4a4*^m1Btlr^ mice (at the age of 5 months) have elevated thresholds in ABR (70 dB vs. 48–64 dB in wild type) [25]. No data currently exist for the *Col4Δ3-4* and for the two *Col4a5*-based models.

## 10. Conclusions

Most of our current knowledge in mouse models comes from research stemming from the two *Col4a3* knockout models, presumably due to the early onset of disease phenotype and their simplicity in data interpretation. However, since the complete absence of collagen IV is responsible for only a subset of Alport patients, this raises the possibility of a bias in driving conclusions. This can be seen in the fact that hearing defects have not been detected in *Col4a4*-based models, which are characterized by partial expression of collagen IV, but such defects can be seen in *Col4a3* knockout models characterized by a complete absence of collagen IV. Furthermore, we know that *Col4a3* knockout mice not only have kidney, ocular, and hearing defects but also have cardiorespiratory defects [52,53] and upregulated serum fibroblast growth factor 23 (FGF23) at the earliest stages of renal damage, even before the increase in BUN and creatinine [41]. Whether models other than the *Col4a3* knockout also share the same phenotype is still unknown.

Most of the models are characterized by a short lifetime that is affected by their genetic background. Two models are characterized by an extended survival compared to the rest, the heterozygous *Col4a3*^tm1Dec/J^ and the knockin missense model *Col4a3*^p.G1332E^. Because the heterozygous *Col4a3*^tm1Dec/J^ expresses half of the wild-type *Col4a3*, it better represents thin basement membrane nephropathy (TBMN), the carrier state of AS. Thus, up to date, the only model of AS that can be considered as a late-onset Alport model is the *Col4a3*^p.G1332E^, making this model suitable to study events occurring at an older age.

While in most models, collagen IV is absent from the GBM, in three models, it is persistent: the *Col4a4*^bwk^, the *Col4a3*^em1Bghn^, and the *Col4a3*^p.G1332E^. The *Col4a4*-based model is weakly positive for Col4a3, Col4a4, and Col4a5 expression, which can assemble into a trimer. In the *Col4a3*^em1Bghn^, the expression of Col4a3, Col4a4, and Col4a5 chains is normal. This highlights the toxic effect of the Z-appendage on the collagen IV function. In the missense model, the expression of cleaved fragments of Col4a3, Col4a4, and Col4a5 (~35 kD at the NC1 domain) is persistent in glomeruli (but not tubules). It is unknown if this fragment exerts any effect on disease phenotype. Nevertheless, the fact that this missense mutation model has a milder disease course and a substantially longer lifespan might indicate that the secretion of even mutant collagen trimers in the GBM is better than null secretion [22]. Col4a3 can be digested by Mmp9 at the carboxyterminal domain to release the 28 kD antiangiogenic and proapoptotic peptide tumstatin [54,55]. Whether the 35 kD fragment has a tumstatin-like activity in *Col4a3*^p.G1332E^ mice remains to be investigated. Importantly, neither intact collagen IV nor this fragment can be detected in the tubules of these mice, suggesting a different molecular regulation between glomeruli and tubules of the *Col4a3*^p.G1332E^. This aligns with the differences in phenotype initiation/progression between glomeruli and tubules that are observed in other models.

Through this review, we detected two major unmet needs. First, there is a need to study more models with missense mutations in the different domains of the three collagen chains. Mutations of glycine substitutions at different locations, or mutations involving other amino acids, including crucial prolines, might shed light on more details regarding the molecular and cellular pathomechanisms of AS, thereby advancing our understanding. The second pressing need is for more studies concerning hearing loss and ocular abnormalities, which would enable the development of potential treatments.

In addition, it turns out that the minimum phenotypic analysis of AS mice should include not only the analysis of glomerular structure/function (for example, GBM structure, hematuria, proteinuria, and glomerular filtration rate) and lifespan but also the analysis of the tubular structure and function. This is emphasized by recent findings, which show that depending on the background, tubular alterations can precede glomerular alterations [27]. The expression of KIM-1 and MCP-1 can be used as biomarkers of kidney tubular defects. Importantly, a recent study suggests MCP-1 as a biomarker of renal decline in AS patients [27,56]. In closing, we mention a recent elegant work in which they studied a *Col4a3* knockout mouse (such as the *Col4a3*^tm1Jhm^) together with a new *Col4a5* knockout model (deletion of exon 36, obtained from the International Mouse Knockout Consortium) [57]. Both models are in the C57BL/6N strain. The authors report a thickened GBM, a progressively increased albumin-to-creatinine ratio, and podocyte foot process effacement in both models. Sclerosis occurred in *Col4a5*−/− mice at 16 weeks of age. Importantly, this study demonstrates that the glomerular matrix composition is altered in the two models in an age- and genotype-dependent manner, even before the appearance of ultrastructural changes. An increased abundance of proteins involved in cell-matrix adhesion (for example, in integrin adhesion components and their ligands) in Alport models is reported along with a reduction in metabolic and mitochondrial components. These findings agree with the findings of another study using the *Col4a3*^tm1Dec/J^ and primary tubular cell cultures [44].

Finally, we should mention a most recent mouse model created to recapitulate thin basement membrane nephropathy caused by null mutations in the prolyl 3-hydroxylase 2 (*P3h2*) gene, which hydroxylates 3′-prolines of the collagen IV alpha chains (Gly-3Hyp-4Hyp-Gly). Conditional podocyte knockout animals lacking gene expression expressed a collagen IV glomerulopathy, which included thin basement membranes and abnormalities reminiscent of AS [57]. This publication demonstrated that the prolyl-hydroxylation is not of decorative nature but of essential functional significance [58].

## Figures and Tables

**Table 1:** Existing mouse models of Alport syndrome and their genetic alterations. MGD: Mouse Genome Database (www.informatics.jax.org); ES cells: embryonic stem cells.

Date	Type of Genetic Modification/Alteration	Description of the Specific Alteration	Official Name in MGD	Existence in Humans	Reference
1996	Knockout (in R1 ES cells)	*Col4a3* Neo cassette replaced the first three exons of the NC1 domain	*Col4a3* ^tm1Jhm^	No	[18]
1996	Knockout (in 129X1/SvJ ES cells)	*Col4a3* Neo cassette replaced exon 5 of the NC1 domain	*Col4a3* ^tm1Dec/J^	No	[19]
2006	Heterozygous knockout	*Col4a3* Neo cassette replaced exon 5 of the NC1 domain	*Col4a3*^tm1Dec/J^/+	No	[20]
2010	Knockout of mouse Col4α3 NC1—Knockin of human Col4α5 NC1	*Col4a3* Substitution of the mouse Col4α3 NC1 with the human Col4α5 NC1	*Col4a3* ^tm1.1Rk1^	No	[21]
2014	Homozygous knockin—missense mutation	*Col4a3* Substitution p.G1332E	Not existing yet—here, presented as *Col4a3*^p.G1332E^	Yes	[22,23]
2014	Heterozygous knockin—missense mutation	*Col4a3* Substitution p.G1332E	Not existing yet—here, presented as *Col4a3*^p.G1332E^/+	Yes	[22,23]
2021	Compound heterozygous knockin—missense mutation	*Col4a3* Substitution p.G1332E Created by crossing the homozygous *Col4a3*^tm1Dec/J^ with the homozygous *Col4a3*^p.G1332E^	Not existing yet—here, presented as *Col4a3*^p.G1332E^/-	Yes	[22,23]
2021	Homozygous CRISPR/cas-mediated knockin of a peptide	*Col4a3* Substitution of His1669 with an 8 amino acids peptide (QQNCYFSS—Z-appendage)	*Col4a3* ^em1Bghn^	Yes	[24]
2021	Heterozygous CRISPR/cas-mediated knockin of a peptide	*Col4a3* Substitution of His1669 with an 8 amino acids peptide (QQNCYFSS—Z-appendage)	*Col4a3*^em1Bghn^/+	Yes	[24]
2011	ENU mutagenesis, single nucleotide mutation—destroys splice donor site	*Col4a4* GA in the intron 8 destroying the canonical splice donor site	*Col4a4* ^m1Btlr^	No	[25]
2013	Spontaneous mutation	*Col4a4* GA in the GT splice donor of exon 30—exon skipping but maintains ORF	*Col4a4* ^bwk^	No	[26]
2019	ENU-mediated mutagenesis	*Col4a4* Nonsense mutation p.G400X	*Col4a4* ^m1H^	No	[27]
1999	Double knockout	*Col4a3* and *Col4a4* Insertion of the 4.1kb tyrosinase minigene TyBS	Not existing yet—here, presented as *Col4Δ3-4*	No	[28]
2004	Single nucleotide mutagenesis	*Col4a5* Nonsense mutation c.213 G>T; p.G5X; creation of a stop codon at exon 1 (7S domain)	*Col4a5* ^tm1Yseg^	Yes	[29]
2019	Single nucleotide mutagenesis (CRISPR/cas)	*Col4a5* Nonsense mutation c.1411 C>T; p.R471X; creation of a stop codon at exon 21	*Col4a5* ^em1Keha^	Yes	[30]

**Table 2:** Lifespan and kidney-related phenotypic features in mouse models of Alport syndrome. GBM: glomerular basement membrane; TBM: tubular basement membrane; RBCs: red blood cells.

Strain	Model’s Name	Lifespan	Hematuria	Proteinuria	Fibrosis—Inflammation	GBM Morphology/ Composition	Glomerular Alterations	Tubular Alterations	Expression of Collagens	Serum Creatinine	BUN/ Serum Urea	Reference
(129X1/SvJ x 129S1/Sv)F1-Kitl^+^	*Col4a3* ^tm1Jhm^	3 months (5% lived longer than 4 months)	Absent	Protein/creatinine ratio: 2–>20-fold after 2 months	Extracellular matrix between tubules by ~P60	Altered appearance (basket weave-like) and molecular composition by P60	Thickened Bowman’s capsule, thickened-closed capillary loops, filled with hyalin by P60	Atrophied, dilated, filled with hyalin by ~P60, Kim-1 upregulation, apoptosis	Absence of Col4a3, Col4a4, and Col4a5 but presence of Col4a1, Col4a2, and collagen VI in GBM	Normal until 2 months, then increased	Normal until 2 months, then increased	[18,43]
129X1/SvJ	*Col4a3* ^tm1Dec/J^	50% survival: 70.9 ± 6.0 days	Present (900–3000 corpuscles per mL) by 2 weeks	10-15 mg/ml by 6-6.5 weeks	Present in glomeruli by 14 weeks	Focal thinning, splitting, progressing rarefication, and multilamination) and altered molecular composition at 4 to 14 weeks	Thickened Bowman’s capsule, expansion of the mesangial matrix, presence of microvilli on the pedicles, collapsed capillaries	Enhanced staining for fibronectin, increased proliferation, metabolic, and mitochondrial defects	Absence of Col4a3, Col4a4, and Col4a5 from GBM and TBM and the presence of Col4a1 and Col4a2 into mesangial area, GBM, and TBM	Increased at 8–9 weeks	Began to rise at ~10 weeks; reached 10x of wild-type control	[19,20,44]
129X1/SvJ	*Col4a3*^tm1Dec/J^-heterozygous	50% survival Col4a3^+/−^: 21.7 ± 2.5 SD months; Col4a3 ^+/+^: 30.3 ± 2.4 SD months; Col4a3 ^−/−^: 70.9 ± 6.0 SD days	Present after 8 weeks	Mild proteinuria (>0.1 g/L) increased to >3 g/L before death	Upregulated expression of fibronectin and fibrosis markers at 10 and 30 weeks	Reduction in the thickness of GBM by 12 months	Increased mesangial cells, thickened Bowman’s capsule, expression of EHS- laminin, interstitial myofibroblasts, and macrophages in glomeruli at 30 weeks, glomerulosclerosis at 12 months	Increased cellular size, vast amounts of euchromatin, increased intratubular protein load at 12 months	Not measured	Not measured	Increased at 18 months; reached >125 mg/dL before death	[20]
C57BL/6J (10+ generations from 129Sv to C57BL/6J)	*Col4a3* ^tm1.1Rk1^	23–30 weeks	Not measured	Albumin/creatinine ratio: 0.82 ± 0.16 at 8 weeks; 17.37 ± 0.83 at 12 weeks	Interstitial fibrosis/inflammatory infiltration initiating at 12 weeks; extensive at 22 weeks (61% ± 5.31)	Altered molecular composition with Col4a1/Col4a2 chains assembling into the α2α1α1/α2α1α1 network; focal thinning-thickening at 22 weeks	Podocyte foot process effacement at 22 weeks; glomerulosclerosis initiating at 12 weeks; extensive at 22 weeks (38% ± 3.6)	Tubular atrophy (initiating at 12 weeks; extensive at 22 weeks)	Absence of Col4a3 and Col4a4 from GBM and the presence of Col4a1, Col4a2, Col4a5, and Col4a6 into mesangium, GBM, and Bowman’s capsule	Increased at 22 weeks (0.425 ± 0.0692 mg/dL)	Not measured	[21]
129X1/SvJ (5 generations from C57BL/6J to 129X1/SvJ)	*Col4a3*^p.G1332E^ homozygous	Mean survival time: 15.1 months	Intermittent after 3 months	Albuminuria: >0.1 g/L at 3 months, increased to >1 g/L after 15 months	Moderate to severe periglomerular and interstitial fibrosis; mild to moderate infiltration by lymphocytes by 20 months	Thinning-thickening and splitting morphology by 5 months	Segmental or global glomerulosclerosis by 20 months	Tubular injury	Expression of ~35 kDa collagen IV NC1 fragments at the GBM, absence at the TBM	Increased at 15–22 months in 62.5% of homozygotes	Increased at 15–22 months in 62.5% of homozygotes	[22]
C57BL/6J	*Col4a3* ^em1Bghn^	Not measured	Not measured	Moderate albuminuria (albumin-to-creatinine ratio) from 9 to 23 weeks	Not measured	GBM thinning and thickening, lamellated and occasionally split (age not defined)	Podocyte foot process effacement (age not defined), glomerulosclerosis; occasional formation of crescents	Not measured	Normal expression of Col4a3, Col4a4, and Col4a5 proteins	Not measured	Not measured	[24]
C57BL/6J	*Col4a3*^em1Bghn^ heterozygous	Not measured—some mice were used for experiments at the age of one year	Not measured	Mild albuminuria (albumin-to-creatinine ratio) at one year	Not measured	GBM thinning and thickening at one year	Glomerulosclerosis	Not measured	Normal expression of Col4a3, Col4a4, and Col4a5 proteins	Not measured	Not measured	[24]
C57BL/6J	*Col4a4* ^m1Btlr^	6–7 months	Present by 3 months old (Chemstrip analysis)	Proteinuria initiates at 3 months, established by 4 months (Chemstrip analysis)	Focal/segmental glomerulosclerosis; Interstitial fibrosis by 5 months	Not examined	Not examined	Tubular atrophy and dilation by 5 months	Not measured	Not measured	Increased at 5 months	[25]
NONcNZO4/Lt	*Col4a4* ^bwk^	Mean survival time: 124 days	Not measured	Elevated Albumin/creatinine by 4 weeks, reaching 3,400 mg/g at 6 weeks	Glomerulosclerosis, tubulointerstitial nephritis, inflammatory cells in the interstitium at 3 months	Not examined	Synechiae, expansion of mesangial matrix, glomerular crescents at 3 months	Tubular protein casts, tubular atrophy examined at 3 months	Low expression of Col4a3/a4/a5 and increased expression of Col4a2 in the GBM at 5 and 10 weeks	Not measured	Not measured	[26]
129S1/ SvImJ		Not measured	Not measured	Albumin/creatinine 3000 mg/g (females), 5000 mg/g (males) at 12 weeks	Late-onset (9 weeks) glomerulosclerosis, inflammatory cell infiltration	Splitting, thickening, and basket-weave-like morphology, extensive lesions at 6 weeks	Podocyte foot effacement at 6 weeks	Late-onset (9 weeks) tubular protein casts and tubular atrophy	Not measured	Not measured	Not measured	
DBA/2J		Mean age of death: 84 days	Not measured	Albumin/creatinine 7300 mg/g (females), 8700 mg/g (males) at 12 weeks	Early-onset (6 weeks) glomerulosclerosis and mild inflammatory cell infiltration	Splitting, thickening, basket-weave-like morphology, extensive lesions at 6 weeks	Podocyte foot effacement at 6 weeks	Early-onset (6 weeks) tubular protein casts, tubular atrophy	Not measured	Not measured	Not measured	
63% C3H.Pde6b+/37% C57BL/6J	*Col4a4* ^m1H^	Mean survival time: 70 days	Not measured	Protein/creatinine ratio: not significantly increased at 4 and 7 weeks		Not examined	Glomerular enlargement, increased cellularity, and sclerosis (at 53 days)	Tubular dilation, hyaline casts (at 53 days)	Absence of Col4a4 from glomeruli	Increased at 4 weeks	Increased at 4 weeks	[27]
C3H.Pde6b+ enriched		Mean survival time: 47 days	Present at 4 weeks (dipstick analysis)	Present from 4 weeks (dipstick analysis)	Reduced inflammation markers by 4 weeks in the B6-enriched strain compared to the C3H-enriched strain		4 weeks: larger-swollen podocytes, thickened foot processes, flattened, and irregular. 7 weeks: podocyte effacement	Increased expression of (Kim-1) by 4 weeks		Increased at 7 weeks	Increased at 7 weeks	
C57BL/6J enriched		Mean survival time: 97 days	Not present at 4 weeks (dipstick analysis)	Present from 7 weeks (dipstick analysis)			No glomerular alterations at 7 weeks	Hyaline casts, mild tubular basophilia at 49 days. Reduced Kim-1 expression		Normal at 7 weeks	Normal at 7 weeks	
FVB/N	*Col4* *Δ3-4*	10-14 weeks	Present at 2 weeks	Detectable at 2 weeks; 10-fold elevated at 1 month	Interstitial fibrosis by 8 weeks	Thin/focally duplicated at 2 weeks. Thicker/disorganized with basketweaving of the lamina densa later	Hyperplasia of parietal epithelial cells; crescentic glomerulonephritis; increased mesangial or endocapillary cells; BrdU positivity of parietal and endocapillary cells at 5 weeks	Intratubular RBCs; protein casts at 2 weeks. Tubular cells BrdU positive at 4 weeks. Tubular injury and tubular atrophy at 8 weeks	Col4a3, Col4a4, and Col4a5 absent from both GBM and TBM. Col4a1 and Col4a2 are detected in the GBM, TBM, and mesangium	Normal at 6 weeks 10-fold elevated at 12 weeks	Not measured	[28]
C57BL/6	*Col4a5* ^tm1Yseg^	From 6 to 34 weeks; median: 23 weeks (males); From 8 to 45 weeks; median: 39 weeks (female carriers)	Not measured	Present in males after 7 weeks; Proteinuria in female carriers after 9 weeks	Males at 17 weeks: widespread interstitial inflammation and focal sclerosis in glomeruli	Males: lamellation at 4 weeks, lamellation-splitting at 17 weeks, podocyte foot process effacement, vesiculation, and denudation; Females: lamellation at 17 weeks	Males: at 4 weeks, capillary wall thickening, mesangial hypercellularity. At 7 weeks, capillary loop dilation, capillary tuft collapse, capsular adhesions. Females: at 17 weeks, focal abnormalities	Males: at 4 weeks, sparing of tubulointerstitium. Females: at 17 weeks, focal abnormalities	Loss of Col4a3, Col4a5 from GBM and TBM and of Col4a6 from Bowman’s capsule; conserved expression of Col4a1 and Col4a2. Mosaic Col4a5 and Col4a3 in females	Not measured	Increased plasma urea nitrogen levels	[29]
C57BL/6J	*Col4a5* ^em1Keha^	Hemizygous males: started dying at 26 weeks; 72.2% died by 30 weeks. Median: 28 weeks	Present after 22 weeks	Present and increasing before 10 weeks	Interstitial fibrosis by 6 weeks	At 6 weeks: focal irregularity of GBM/occasional foot process effacement; at 22 weeks: marked thickening with matrix lamination in GBM	Glomerular tuft collapse thickened Bowman’s capsule. Parietal cell hyperplacia and increased mesangial matrices at 6 weeks. Glomerulosclerosis at 22 weeks	Tubulointerstitial changes associated with glomerulosclerosis initiating at 6 weeks	Loss of Col4a5 from GBM and TBM at 6 weeks	Increased levels after 10 weeks	Increased levels after 10 weeks	[30]

**Table 3:** Ocular and hearing defects occurring in the mouse models of Alport syndrome.

Model Name	Hearing Loss	Ocular Abnormalities	Reference
*Col4a3* ^tm1Jhm^	Increased auditory threshold in 3/12 mutant mice (at P89 and P96)	Not measured	[18]
*Col4a3* ^tm1Dec/J^	Structural/biochemical alterations, alterations in cochlear basement membrane, increased thickness of the cochlear basement membrane, increased auditory threshold	Irregular interior layer of basement membrane encasing the anterior lens, reduced anterior capsule apical angle, increased thickness of the basement membrane of retinal capillaries	[19,44,51]
*Col4a3*^tm1Dec/J^-(heterozygous)	Not measured	Not measured	[20]
*Col4a3* ^tm1.1Rk1^	Not measured	Not measured	[21]
*Col4a3*^p.G1332E^ (this entry refers to all three models with the glycine substitution to glutamate)	Not measured	Not measured	[22]
*Col4a3*^em1Bghn^ (this entry refers to both the homozygous and the heterozygous models)	Not measured	Not measured	[24]
*Col4a4* ^m1Btlr^	Elevated thresholds in 2/6 mice aged 5 months old	Not measured	[25]
*Col4a4* ^bwk^	No deviations in hearing were found (age not defined)	No ocular histological alterations	[26]
*Col4a4* ^m1H^	No hearing loss (examined at 6–7 weeks)	No alterations in optokinetic drum scores, slit lamp, and ophthalmoscopic observations (examined at 6–7 weeks)	[27]
*Col4* *Δ3-4*	Not measured	Not measured	[28]
*Col4a5* ^tm1Yseg^	Not measured	Not measured	[29]
*Col4a5* ^em1Keha^	Not measured	Not measured	[30]

## Data Availability

Not applicable.
